# The Role of Osteopontin in Psoriasis—A Scoping Review

**DOI:** 10.3390/jcm13030655

**Published:** 2024-01-23

**Authors:** Agnieszka Kimak, Anna Woźniacka

**Affiliations:** Department of Dermatology and Venereology, Medical University of Lodz, Hallera 1, 90-647 Lodz, Poland; agnieszka.kimak@umed.lodz.pl

**Keywords:** comorbidities, osteopontin, psoriasis

## Abstract

Psoriasis is a chronic systemic disease with an immunological basis and a complex pathophysiology. The chronic inflammatory status of psoriasis is associated with several comorbidities, such as metabolic syndrome, obesity, and cardiovascular disease. The development of psoriasis is influenced by osteopontin, a glycoprotein that influences physiological and pathological reactions by modulating Th1 and Th17 cellular responses, stimulating keratinocyte proliferation, regulating cellular apoptosis, and promoting angiogenesis. The recent identification of immune pathways involved in psoriasis development has facilitated the development of biological treatments; however, a better understanding of the intricate relationship between underlying inflammatory processes, psoriasis development, and accompanying comorbidities is needed for improved disease management.

## 1. Introduction

Osteopontin (OPN) is a glycoprotein expressed in many tissues and cells. The *Opn* gene is located on chromosome 4 and belongs to the small integrin-binding ligand N-linked glycoprotein (SIBLING) family [1]. After transcription and translation, the protein is phosphorylated, glycosylated, and modulated by proteolytic cleavage, which results in different isoforms of the molecule—secreted OPN (sOPN) and intracellular OPN (iOPN), as well as their different functionalities after binding with different receptors and cells [2].

The effects of OPN can be context-dependent, influenced by the tissue microenvironment, the presence of other molecules, and the engagement of specific receptors. In pathological conditions, such as inflammation or cancer, elevated OPN expression may contribute to disease progression, while in normal physiological conditions, it plays roles in tissue homeostasis and repair. For example, in apoptosis regulation, OPN emerges as a vital anti-apoptotic factor, preventing programmed cell death in macrophages, T cells, fibroblasts, and endothelial cells exposed to harmful stimuli [3]. OPN’s involvement in biomineralization is marked by its inhibition of crystal growth in calcium-based biominerals, such as bones and teeth [4]. In bone remodeling, OPN may anchor osteoclasts to bone surfaces, facilitating cell attachment and migration, while also initiating the process leading to bone resorption [5]. The impact of osteopontin on cell activation is manifested in its ability to modulate T cell differentiation, favoring Th1 responses while inhibiting the production of Th2 cytokines. It influences cell-mediated immunity, enhances B cell function, and induces mast cell degranulation. Furthermore, OPN plays a crucial role in chemotaxis, cell adhesion, migration, and survival within the immune system, making it a pivotal player in immune modulation and inflammatory responses [6].

The expression and function of OPN in healthy skin are not fully known. OPN was found in the basal cell layer, hair follicles, sebaceous, and sweat glands [7]. In innate immunity, OPN acts as a chemoattractant and as an enhancer of the phagocytic activity of macrophages. It promotes the production of proinflammatory cytokines, such as interferon-g (IFN-g) [8]. In an adaptive immune system, OPN promotes the differentiation of T helper 1 cells (Th1) and T helper 17 cells (Th17) through the stimulation of interleukin 12 (IL-12) and interleukin 17 (IL-17) production and the suppression of interleukin 27 (IL-27) [9,10,11]. OPN overexpression is associated with many dermatological conditions, including autoimmune diseases, allergies, granulomatous diseases, malignancies, and infections [6].

**Figure 1:**
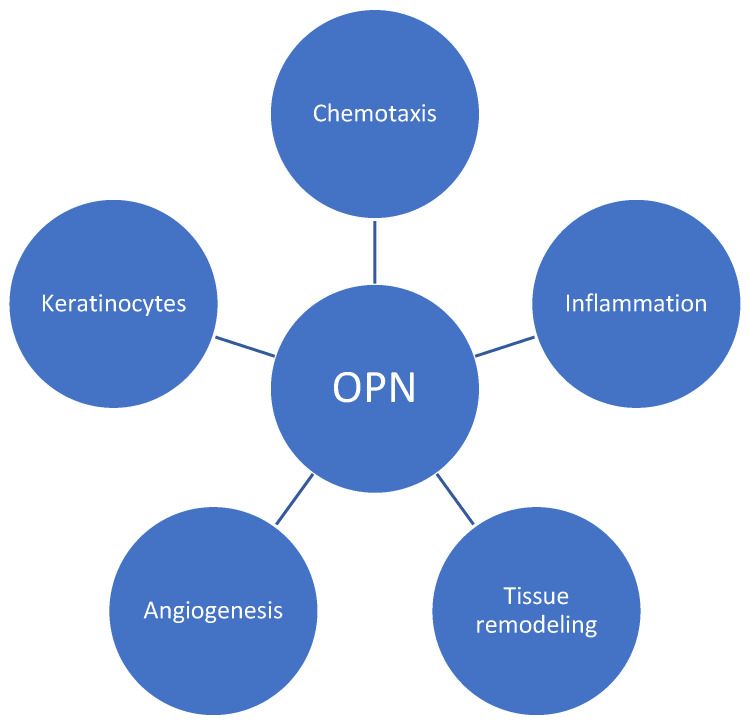
Role of osteopontin (OPN) in psoriatic plaque development. OPN regulates T-cell differentiation, favors Th-1 responses, and influences various immune cell responses. By utilizing integrins and signaling cascades, it induces chemotaxis and contributes to tissue remodeling via its impact on metalloproteinases. Additionally, OPN promotes angiogenesis and enhances the proliferation of keratinocytes.

OPN plays a role in the pathogenesis of psoriasis in a variety of manners, including chronic inflammation and aberrant skin cell proliferation characteristics of the disease (Figure 1). It modulates T-cell-mediated immunity by inducing the effects on Th17 and Th1 using its intracellular and secreted variant forms, respectively [6]. According to oncology studies, OPN can bind to several integrins, including αvβ1, αvβ3, and αvβ5 [12]. These interactions initiate signaling cascades (including focal adhesion kinase (FAK), mitogen-activated protein kinase (MAPK), and phosphoinositide 3-kinase (PI3K) pathways), leading to the reorganization of cytoskeleton in immune cells, which is crucial for the migration and movement of cells—a process called chemotaxis [13,14,15]. A similar effect may contribute to psoriasis development. In addition, fibroblasts and keratinocytes may be influenced by osteopontin through matrix metalloproteinases (MMPs) and tissue inhibitors of metalloproteinases (TIMPs), contributing to abnormal tissue remodeling observed in psoriatic skin [16,17]. Finally, sOPN promotes enhanced epidermal proliferation via keratinocyte apoptosis inhibition and is also associated with vascular changes leading to increased angiogenesis in psoriatic lesions [18].

## 2. Materials and Methods

**Figure 2:**
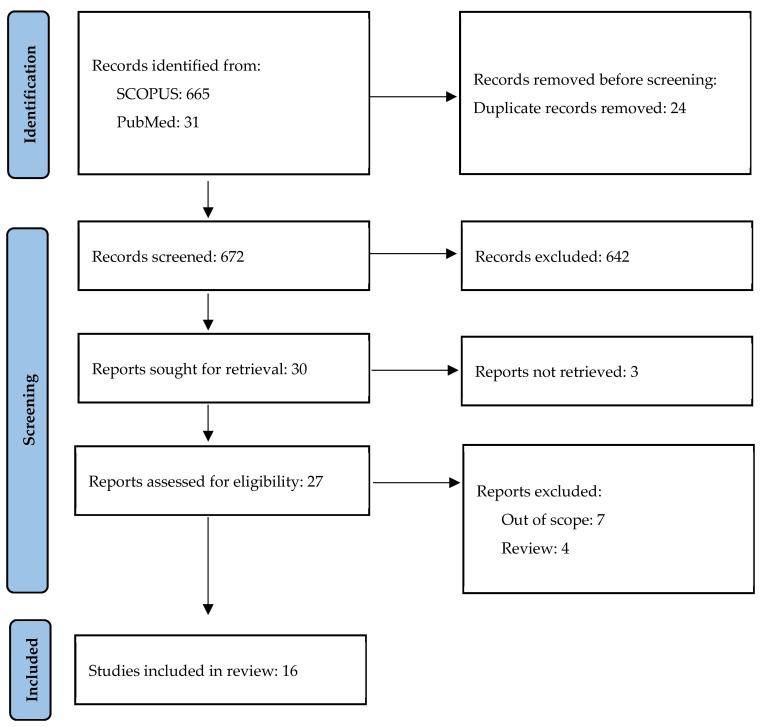
Study selection.

A study on the role of OPN in psoriasis was conducted on 25–31 October 2023 by AK. The search included MeSH terms “psoriasis” and “osteopontin” in articles written in English or Polish in the SCOPUS and PubMed databases; in total, 696 potentially eligible articles published since the year 2000 were detected. After the removal of duplicates and irrelevant articles based on the titles and abstracts, 16 original articles were included in this review (Figure 2 and Table 1).

## 3. Review Summary

### 3.1. Osteopontin and Metabolic Syndrome Components

In all 16 evaluated articles, there was a higher expression of OPN in the psoriasis (PsO) group than in the healthy control (HC) groups. Twelve groups measured serum OPN [17,19,20,21,22,23,24,25,26,27,28,29], four reports investigated tissue OPN [7,18,23,30], and the expression of OPN genotypes and mRNA in peripheral blood mononuclear cells was evaluated in three studies [24,29,31]. The correlation between serum and tissue OPN was investigated in one paper and no correlation was found [23]. Mean serum OPN levels were diverse and incomparable between the studies. This may be explained by no standard methodology and the lack of laboratory standards.

Many authors have tried to correlate OPN serum concentrations with metabolic syndrome (MetS) and its components. The results were contradictory. While some researchers found higher OPN concentrations in PsO with Mets [22,24,29], others found no correlation between plasma OPN and MetS frequency [22,23,24,25,29]. Some MetS components, such as body mass index (BMI), diabetes mellitus (DM), insulin resistance (IR), and hypertension (HT), positively correlated with OPN [19,24]. Total cholesterol (TC) and triglycerides (TGs) had contrary results [19,25]. OPN’s multifaceted roles in relation to key aspects of metabolic syndrome, including obesity, insulin resistance, inflammation, vascular dysfunction, and dyslipidemia, have been studied in recent years, but still remain elusive. OPN expression in adipose tissue may contribute to the chronic low-grade inflammation characterizing adipose tissue in obesity, promoting insulin resistance by interfering with insulin signaling between adipocytes and peripheral tissues and by promoting the accumulation of macrophages in adipose tissue [32]. In the realm of vascular dysfunction, OPN’s role in endothelial dysfunction and atherosclerotic plaque formation has been explored as well. OPN was found to be independently associated with the severity of coronary atherosclerosis and with increased risk for major adverse cardiac events [33]. Finally, OPN was found to be implicated in non-alcoholic fatty liver disease (NAFLD) progression by enhancing hepatic inflammation and fibrosis [34]. In summary, despite extensive research enhancing our comprehension of osteopontin’s involvement in crucial facets of metabolic syndrome, the current state of knowledge does not permit a precise definition of its specific contribution to these processes.

Among reviewed articles, a few cardiovascular biomarkers, such as C-reactive protein (CRP), chemerin elevation, and fetuin-A decrease, were found in PsO patients [27]. Common carotid artery intima–media wall thickness, a marker of subclinical atherosclerosis, was also greater in PsO and correlated positively with serum OPN [19]. Adiponectin, a core homeostatic factor involved in glucose and lipid metabolism, was found to be lower in PsO and negatively correlated with OPN, BMI, and MetS as well [22]. This corresponds with previous research that has emphasized the effect of adiponectin in psoriasis-related comorbidities and plaque formation. Adiponectin was found to be involved in the pathogenesis of PsO through its anti-inflammatory effects and the inhibited production of, e.g., IL-2, IL-6, IL-8, IL-17, IL-22, tumor necrosis factor-α (TNF-α), and interferon-γ (IFN-γ), as well as the stimulated production of IL-10 [35,36]. A lower level of adiponectin may worsen the course of psoriasis and influence the treatment efficacy.

### 3.2. Osteopontin and Psoriasis Severity

Only two papers found a positive correlation between the psoriasis severity area index (PASI) and lesional or plasma OPN [22,30]. Outcomes from other research did not confirm the correlation between the PASI and OPN [19,20,21,23,24,26,28]. No correlation between PsO duration and OPN was found [19,20,21,23,24,30]. There was no correlation between OPN concentration and the age of onset of psoriasis or a positive family history of PsO [23]. A higher concentration of OPN in older patients was found in two studies [19,20], but this finding was not observed in other research [22,23].

### 3.3. Osteopontin and Interleukin-17

The correlation between osteopontin and interleukin-17 (IL-17) was investigated by Przepiórka-Kosińska’s team [26]. Both serum OPN and interleukin-17 (IL-17) serum levels were elevated in the examined PsO group, but there was no significant correlation between their concentrations [26]. OPN was found to favor the differentiation of T cells into Th1 and Th17 and may also directly stimulate Th17 to produce IL-17 by binding with integrin αvβ3 [6,37]. However, OPN modulates different immune cells and acts through various cytokines; hence, the relationship between OPN and other cytokines may be more complex. As an illustration, a study exploring the role of osteopontin (OPN) in multiple sclerosis (MS) revealed elevated levels of OPN and IL-23 in MS patients. However, it is noteworthy that no significant correlation was observed between these molecules in the study [38]. The crosstalk between OPN and other proinflammatory cytokines remains unclear, necessitating additional research to be specifically designed and carried out for this purpose.

### 3.4. Osteopontin and Oxidative Stress

A wide variety of oxidative stress markers were found to be elevated in PsO patients, some of which are known to correlate with disease severity and duration. It is therefore possible that oxidative stress, induced by reactive oxygen species production, may be involved in the initiation and development of psoriasis and its comorbidities [39]. Interestingly, however, one study found PsO patients to have a lower total oxidant status (TOS) than the HC group; despite this, no difference in the total antioxidant status (TAS) or oxidative stress index (OSI) was found between the groups. The study revealed no correlation between the PASI and the OSI. Regarding OPN and oxidative stress markers, no significant relationship was identified [28]. As this aspect lacks prior investigation, further analysis is required to help us understand the association between OPN and oxidative stress. Employing more sensitive molecules, like peroxidation markers, may provide additional insights.

### 3.5. Osteopontin and Selenium

Some studies also investigated the relationship between selenium (Se) and serum OPN and PsO, but with inconsistent results. Meanwhile, Kadry and Rashed found selenium levels to be lower and negatively correlated with plasma OPN and BMI in a group of 20 PsO patients [23]. These results were not replicated by Toossi et al. on another group twice the size [20].

While patients with plaque psoriasis have lower levels of serum Se than healthy populations, they do not benefit from Se supplementation [40,41,42]. Interestingly though, selenium may play a role in obesity development. One study from 2021 found lower levels of selenium in the nails and urine of overweight and obese people [43]. Selenium regulates carbohydrate and lipid metabolisms, and changes in Se levels may contribute to metabolic disorders, mainly through the regulation of glutathione peroxidase activity [44]. Selenium supplementation could aid weight loss, change body composition, and decrease leptin levels if combined with a hypocaloric diet [45]. This subject is still controversial, but Se supplementation may be considered in obese PsO patients.

### 3.6. Osteopontin during Psoriasis Treatment

An Egyptian study compared three psoriasis treatments, viz. psoralen plus ultraviolet A (PUVA), methotrexate (MTX), and cyclosporin A (CsA), on groups of 12 patients. All patients responded well to the treatment, and had a decreased PASI and lesional OPN expression after three months. The greatest PASI reduction was noted in the CsA group, and the greatest OPN decrease was noted in the PUVA group [30]. Plasma and tissue OPN levels were also measured in patients undergoing 24 weeks of anti-TNF-a therapy (adalimumab or etanercept); at the end of the treatment, OPN was significantly reduced and, interestingly, plasma OPN was lower compared to the healthy control group. Moreover, among the healthy volunteers, OPN in peripheral blood mononuclear cells was directly stimulated by tumor necrosis factor alpha (TNF-a) and downregulated by anti-TNF-a molecules. A similar observation was noted for matrix metalloproteinase-9 (MMP-9) [17]. In PsO patients, MMP-9 is overexpressed in neutrophils and increases vascular endothelial cell activation. This causes endothelial dysfunction and thus enhanced vasodilation and vascular permeability, especially in papillary vessels [46]. This vascular remodeling is crucial in the initial phase of PsO and may possibly be a target of future pharmacological interventions.

### 3.7. Osteopontin in Psoriatic Lesions and Healthy Skin

Although OPN concentration is known to be elevated in psoriatic plaques, it may be surprising to learn that this is also the case in non-lesional skin in PsO patients. Such OPN overexpression in seemingly healthy skin may explain Koebner phenomenon and the constant readiness of the skin to promote plaque formation [18,23,31]. The OPN distribution in psoriatic skin differs from that noted in healthy controls. In healthy skin, OPN is usually expressed in the basal cell layer, hair follicles, sebaceous glands, and sweat glands, while in lesional skin, it can be found at all levels of the epidermis in the inflammatory cells and in the endothelial cells of the dermis [7]. Immunochemical studies found higher Ki-67 and CD34 expression levels in both lesional and non-lesion skin, and found their levels to be correlated. Moreover, OPN and CD34 were also positively correlated with each other [7]. Since Ki-67 is a marker of proliferation and CD34 is an angiogenesis marker, these findings confirm that these processes have important roles in psoriatic plaque formation.

### 3.8. Osteopontin Genotype

A comparison of osteopontin allele distributions in 268 DNA samples from PsO patients with 146 DNA samples from a control group found no difference between PsO and HC; the analysis was based on six single-nucleotide polymorphisms (OPN 616G/T, 443T/C, 302A/C, 156 ins, 1083A/G, and 1239C/A) [24]. Abdel Hay et al. report significant differences in the frequencies of various OPN alleles in PsO compared to healthy subjects [29]. In a much smaller group of 12 PsO patients, a higher OPN gene expression was noted in the lesional skin and PBMC of PsO patients [17]. It is still unclear whether OPN polymorphisms increase the risk of psoriasis development.

## 4. Osteopontin and Other Soluble Biomarkers Related to Other Psoriasis Comorbidities

Psoriasis is associated with many comorbid conditions outside the MetS group, and specific biomarkers can be used to detect and monitoring. These vary in specificity and sensitivity, but may be used as complimentary tools.

### 4.1. Psoriatic Arthritis

Psoriatic arthritis (PsA) affects up to 20% of PsO patients [47]. Apart from genetical and imaging biomarkers, some proteins have been proven to be associated with a greater risk of PsA development and suggested as screening tools.

In the reviewed articles, no correlation between psoriatic arthritis prevalence among psoriatic patients and tissue [23] or plasma [21,23] osteopontin levels was described. However, osteopontin has been identified as one of the key proinflammatory modules in the synovial fluid in PsA patients [48,49]. The objective of the Al-Mossawi group was to examine synovial fluid (SF) and blood leucocytes using mass cytometry and transcriptomic analysis. They observed an expansion of monocytes and macrophages in SF compared to blood. Importantly, these cells exhibited the spontaneous production of osteopontin and chemokine (C-C motif) ligand 2 (CCL2). Gene expression analysis further indicated a significant upregulation of genes associated with osteopontin and CCL2 in psoriatic arthritis (PsA) monocytes/macrophages, with elevated levels of both proteins detected in PsA SF [49]. OPN may promote PsA development by boosting the production of IFNγ and IL-12, diminishing the production of IL-10 and thus fostering the attachment of osteoclasts to the mineralized bone matrix [48].

Other PsA biomarkers include serum cartilage oligomeric matrix protein (COMP), serum matrix metalloproteinase-3 (MMP3), highly sensitive CRP (hsCRP), and osteoprotegerin (OPG) [50,51]. In addition, tissue-resident memory CD8+ T cells (TRMs) originating from the skin may be detected in abundance in the peripheral blood in PsA patients. It is suggested that disturbances in the skin’s immune system contribute to PsA development as well [52,53].

### 4.2. Neurodegenerative Diseases

The possible relationship of psoriasis and neurodegenerative disorders (NDs) has not been established yet. It is suspected that chronic inflammation and oxidative stress promote ND development in PsO patients in direct and indirect ways, but the evidence is rather weak [54]. The improper homeostasis of fatty-acid-binding proteins (FABPs), glutamic acid (GA), and neurofilament light chain (NFL) have been suggested in the pathogenesis of Alzheimer’s disease (AD), Parkinson’s disease, and other NDs. Those molecules play a role in the development of the nervous system and disturbances in their levels may lead to impaired neuronal transmission, neuroinflammation, and neurodegeneration [55,56,57]. Those dementia-related biomarkers in PsO could possibly be influenced by the systematic treatment of psoriasis. In a group of PsO patients treated with acitretin or methotrexate, the serum concentration of FABPs and NFL before treatment was significantly higher than in HC and decreased after 12 weeks of therapy [58].

The role of OPN in NDs has been explored as well, including research focusing on OPN production using microglia in the brains of mice engineered to develop AD [59]. According to a study by Qiu et al., the CD11c+ microglia subset has the capacity to produce osteopontin, and as Alzheimer’s disease advances, osteopontin production has increased. Notably, osteopontin was identified as an inhibitor of the amyloid-beta (Aβ) removal pathway, contributing to the formation of plaques. Conversely, CD11c+ microglia lacking osteopontin were found to play a protective role by facilitating the breakdown of Aβ, thereby safeguarding the brain. In addition, analysis of brain tissue from Alzheimer’s patients demonstrated the correlation between elevated osteopontin levels and the greater severity of dementia. In a proof-of-concept study, the blocking of osteopontin with an antibody in Alzheimer’s mice significantly reduced Aβ plaques by over a half [59]. The findings suggest targeting osteopontin production could be a novel approach to Alzheimer’s treatment, though further research is needed to identify potential drugs for human trials.

### 4.3. Mental Illness

The relationship between psoriasis and stress is bi-directional. On the one side, emotional stress is one of the main psoriasis triggers and, on the other, every exacerbation of the skin lesion leads to phycological discomfort. The interplay between those two conditions is probably caused by immune system dysregulation. One key aspect of this connection is the involvement of similar proinflammatory cytokines. In psoriasis, proinflammatory cytokines, like interleukin-6 (IL-6), TNF-a, and interleukin-1 beta (IL-1β), play a pivotal role in driving the chronic inflammation observed in skin lesions. Concurrently, those same cytokines alter the metabolism of neurotransmitters, such as norepinephrine, serotonin, and dopamine, and can influence mood and emotions [60,61].

Osteopontin (OPN) may also be implicated in the development of mental diseases. Neuroinflammation mediated by OPN appears to contribute to the emergence of depressive behavior in mice, and a reduction in OPN expression through knockdown demonstrated antidepressant effects [62]. Moreover, OPN and IFNy serum levels are elevated in schizophrenia and are associated with severe psychotic symptoms [63]. Interestingly, the role of osteopontin (OPN) in the nervous system may not only be detrimental; rather, through neuroinflammation, OPN has the potential to exert regenerative effects on the tissue [64]. Understanding these mechanisms is key in comprehending the intricate interconnection between immune and neurological systems in stress-related conditions.

### 4.4. Inflammatory Bowel Disease

Patients with PsO are at a higher risk of developing inflammatory bowel diseases (IBDs), such as Crohn’s disease (CD) and ulcerative colitis (UC). This association may be explained by a genetic predisposition, immune system dysfunction, or gut flora abnormalities [65]. IL-17 has been identified in the pathogenesis of both PsO and IBD and has a potential for triggering bowel inflammation in patients treated with IL-17 inhibitors, such as secukinumab [66]. The exact mechanism of anti-IL-17 drugs in IBD remains poorly defined, but it is thought that the decrease in IL-17 concentration leads to a reduction in neutrophile aggregation, and thus may enhance gut bacteria overgrowth [67]. OPN is involved in IBD development as well, and its concentration was found to be correlated with the clinical activity of CD and UC [68]. IL-12 and IL-23 are other common transgressors in PsO and IBD, and the shared genetic susceptibility loci in IL23R and IL12B may denote a genetic link between psoriasis and IBD [69,70]. Finally, patients with PsO display decreased microbiome diversity and their gut flora profile resembles that in inflammatory bowel disease [71]. It is known that certain bacterial taxa can have an influence on immune responses, although the direct link remains to be established [72]. Nonetheless, microbiome alterations could potentially be a target for future therapies in psoriasis.

Several other conditions seem to be comorbid with PsO, but the data are still scarce and detectable biomarkers are unknown. Current evidence shows that patients with PsO require a multidisciplinary approach that focuses on the early identification and treatment of related diseases. Affected patients may show a decreased quality of life, not only due to psoriasis severity, but also existing comorbidities. The possibility of influencing the course of cardiovascular or dementia-related disorders in PsO patients via an early introduction of systemic treatment is promising. Considering these data, the undertreatment of PsO is concerning [73]. It may not only lead to the worsening of skin lesions, but may also result in higher morbidity and mortality rates from comorbidities in these patients.

This review provides a comprehensive summary of current knowledge on the role of osteopontin in psoriasis. However, the results may have been subject to certain limitations—the authors may have missed some articles, since the search was conducted by one person, and book publications were not included.

## 5. Conclusions and Future Directions

Osteopontin, a glycoprotein involved in physiological and pathophysiological processes, has emerged as a potential player in psoriasis. OPN expression increases in serum and the skin and is correlated with other biomarkers involved in PsO development. Previous research allowed the identification of specific molecules associated with PsO and has contributed to the development of targeted treatments, such as monoclonal antibodies, to block these inflammatory processes. Osteopontin, although its role is not fully elucidated, may become a candidate for another type of target therapy in psoriasis and psoriatic arthritis in the future.

## Figures and Tables

**Table 1:** The correlations of serum OPN levels in PsO patients with measured parameters. PsO—psoriasis patients; HC—healthy control.

	Positive Correlation	Negative Correlation	No Correlation
Body mass index (BMI)	Robati et al. [19] Toossi et al. [20]		Duarte et al. [21] Kadry et al. [22] Kadry and Rashed [23]
Metabolic syndrome (MetS)	Kadry et al. [22] Chen et al. [24]		Bartosińska et al. [25] Kadry and Rashed [23]
Total cholesterol (TC)	Robati et al. [19] Toossi et al. [20]	Bartosińska et al. [25]	Kadry et al. [22] Kadry and Rashed [23]
Triglycerides (TGs)	Robati et al. [19] Toossi et al. [20]	Bartosińska et al. [25]	Kadry et al. [22] Kadry and Rashed [23]
High-density lipoprotein cholesterol (HDL)			Bartosińska et al. [25]
Abdominal obesity			Bartosińska et al. [25]
Arterial hypertension (HT)	Chen et al. [24]		Bartosińska et al. [25] Kadry et al. [22] Kadry and Rashed [23]
Diabetes mellitus (DM)	Kadry et al. [22] Chen et al. [24]		
Hyperglycemia			Bartosińska et al. [25] Kadry and Rashed [23]
Insulin resistance (IR)	Kadry et al. [22]		Kadry and Rashed [23]
Waist circumference (WC)			Kadry et al. [22] Kadry and Rashed [23]
Psoriasis area severity index (PASI)	Kadry et al. [22]		Kilinc et al. [22] Przepiórka-Kosińska et al. [26] Robati et al. [19] Toossi et al. [20] Duarte et al. [21] Kadry and Rashed [23] Chen et al. [24]
Duration of the disease			Robati et al. [19] Toossi et al. [20] Duarte et al. [21] Kadry et al. [22] Kadry and Rashed [23] Chen et al. [24]
Age of patient	Robati et al. [19] Toossi et al. [20]		Kadry et al. [22] Kadry and Rashed [23]
Psoriatic arthritis			Duarte et al. [21] Kadry and Rashed [23]
Interleukin 17 (IL-17)			Przepiórka-Kosińska et al. [26]
Fetuin-A		Borsky et al. [27]	
Mean intima–media wall thickness of the common carotid artery (MIMT-CCA)	Robati et al. [19]		
Selenium (Se)		Toossi et al. [20] if PsO and HC groups combined Kadry and Rashed [23]	Toossi et al. [20] if PsO and HC groups were considered separately
Prolactin (PRL)			Toossi et al. [20]
C-X-C motif chemokine ligand 9 (CXCL9)			Duarte et al. [21]
Tumor necrosis factor (TNF)			Duarte et al. [21]
Adiponectin		Kadry et al. [22]	
History of cardiovascular disease (CVD)			Kadry et al. [22] Kadry and Rashed [23]
Smoking			Kadry et al. [22] Kadry and Rashed [23]
Age of onset			Kadry and Rashed [23]
Family history			Kadry and Rashed [23]

## Data Availability

The data that support the findings of this systematic review are available from the following repositories: SCOPUS (https://www.scopus.com) and PUBMED (https://pubmed.ncbi.nlm.nih.gov/). All relevant articles included in this review can be accessed through these databases.
